# Controlling Circadian Rhythms by Dark-Pulse Perturbations in *Arabidopsis thaliana*

**DOI:** 10.1038/srep01533

**Published:** 2013-03-25

**Authors:** Hirokazu Fukuda, Haruhiko Murase, Isao T. Tokuda

**Affiliations:** 1 Department of Mechanical Engineering, Graduate School of Engineering, Osaka Prefecture University, Sakai 599-8531, Japan; 2 PRESTO, Japan Science and Technology Agency (JST), 4-1-8 Honcho Kawaguchi, Saitama 332-0012, Japan; 3 Department of Mechanical Engineering, Ritsumeikan University, 1-1-1 Nojihigashi, Kusatsu, Shiga 525-8577, Japan

## Abstract

Plant circadian systems are composed of a large number of self-sustained cellular circadian oscillators. Although the light-dark signal in the natural environment is known to be the most powerful Zeitgeber for the entrainment of cellular oscillators, its effect is too strong to control the plant rhythm into various forms of synchrony. Here, we show that the application of pulse perturbations, i.e., short-term injections of darkness under constant light, provides a novel technique for controlling the synchronized behavior of plant rhythm in *Arabidopsis thaliana*. By destroying the synchronized cellular activities, circadian singularity was experimentally induced. The present technique is based upon the theory of phase oscillators, which does not require prior knowledge of the detailed dynamics of the plant system but only knowledge of its phase and amplitude responses to the pulse perturbation. Our approach can be applied to diverse problems of controlling biological rhythms in living systems.

Circadian rhythms, as characterized by endogenous period of nearly 24 h, are ubiquitous in almost all living organisms. In plant, the circadian rhythms play important roles in gene expressions, photosynthesis, growth, and many other physiological processes^1^,^2^,^3^. The plant circadian system is composed of a large number of self-sustained cellular oscillators that synchronize with each other to produce a strong output rhythm^4^,^5^,^6^,^7^,^8^,^9^,^10^. Desynchronization of the oscillators diminishes the output circadian rhythm, whereas their resynchronization recovers the rhythm. For healthy growth of the plant, it is essential to maintain synchronized activities of the cellular oscillators so as to sustain the stable rhythm. Precise and ecological control of the circadian rhythm provides a key technology for enhancing the plant growth in artificial environments, *e.g.*, in a closed cultivation system (so-called plant factory) or in a system situated on other planets, where light-dark cycles differ from the 24-h period^2^,^11^,^12^,^13^. Although the light-dark signal as in the natural environment is known as the most powerful Zeitgeber to entrain the cellular oscillators^8^,^14^, its effect is too strong so that all oscillators are reset to the same phase, resulting only in a complete synchronization. It is therefore difficult to set the plant rhythms into various forms of synchrony using the daily light-dark cycles. In contrast, a pulse perturbation such as a short-term injection of a bright pulse induces a phase shifting without completely resetting the phase^15^. Moreover, in studies of controlling synchrony in a system of coupled oscillators^16^,^17^,^18^,^19^, application of multiple pulse perturbations is known to force the collective behavior into various forms such as destruction or recovery of the synchronization^20^,^21^. Whether the pulse injection enhances or destroys the synchrony depends upon the timing of the perturbation, which is thoroughly described by a phase response curve (PRC). If the plant circadian system obeys the theory of phase oscillators^16^,^17^, effect of the pulse perturbation on the plant rhythm can be precisely predicted and designed by using the PRC.

Here we show that the theory of phase oscillators can be well applied to the plant circadian rhythm and demonstrate that the plant system can be successfully controlled by application of multiple pulse trains. Our technique does not require prior knowledge of the detailed dynamics of the plant system but it utilizes only the measurement of the PRC and slight amount of pulse perturbations, which do not destroy fundamental properties of the plant circadian system. The significance of our controlling technique is demonstrated by inducing a circadian singularity, *i.e.*, abolished circadian rhythmicity in response to a strong stimulus^16^, as a result of desynchronized activities of the cellular oscillators.

## Results

### Controlling synchrony in a system of coupled oscillators

**Figure 1:**
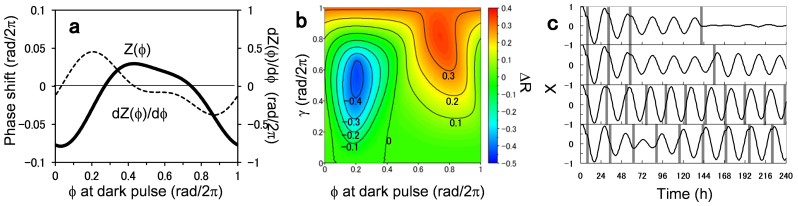
Controlling synchrony of oscillators by pulse perturbations. (a) Phase sensitivity function *Z*(Φ) = −0.093 + 0.327sin(Φ− 1.64) + 0.079sin(2Φ − 2.19) used in our model (Eq. 1) and its slope d*Z*(Φ)/dΦ. The function *Z*(Φ) was determined from PRC *G*(Φ) for the 2-h dark pulse in a real plant as *Z*(Φ) = *G*(Φ − 2π/24)/2. (b) Simulation results showing the effect of pulse perturbation on the mean Φ and range γ of uniformly distributed phase of model oscillators [Φ − γ, Φ + γ]. The pulse effect was computed as Δ*R* = *R*_1_ − *R*_0_ using the circular statistics measured before (*R*_0_) and after (*R*_1_) the application of the 2-h dark pulse. (c) Influence of multiple dark pulses on the mean oscillation amplitude *X* in globally coupled phase oscillators (Eq. 1). The gray bars indicate the timing of the 2-h dark pulses.

Our controlling technique is designed based upon the following model of coupled phase oscillators: 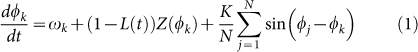
Here, φ_k_ and ω_k_ are the phase and natural frequency, respectively, of the *k*th oscillator, *K* represents the coupling strength, *N* is the number of oscillators, and *L*(*t*) stands for light intensity (*L*(*t*) = 0 for dark pulse; *L*(*t*) = 1 for light on). The phase sensitivity function *Z*(Φ) for the dark pulse is given by *Z*(Φ) = −0.093+0.327sin(Φ− 1.64) + 0.079sin(2Φ − 2.19) (Fig. 1a), which is determined from the PRC^15^ of a real plant (details described in experimental section). In general, the effect of the pulse perturbation on the synchronized oscillators depends upon the slope of the phase sensitivity function^21^. If d*Z*(Φ)/dΦ > 0, the phase difference between two nearby orbits on a limit cycle tends to expand. On the other hand, if d*Z*(Φ)/dΦ < 0, the phase difference between the two orbits tends to shrink. Expansion and contraction of the phase difference leads to suppression and enhancement, respectively, of the synchronized state of the oscillators. Moreover, the effect of the pulse perturbation on the network dynamics depends upon the phase distribution of the oscillators. For a network of oscillators with uniformly distributed phases in the range of [Φ − γ, Φ + γ], the dependence of the effect of a single dark pulse on the mean phase Φ and the distribution range γ was numerically calculated and shown in Fig. 1b. The effect of the pulse was quantified as Δ*R = R_1_ − R_0_*, where *R_0_* and *R_1_* represent values of the circular statistics (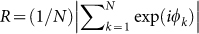) measured before and after the perturbation (*R* indicates the level of synchrony; complete synchrony for *R* = 1 and desynchrony for *R* = 0). At Φ = 0.2 and 0.85 rad/2π, the dark pulse had a strong effect of inducing desynchronization and synchronization, respectively, agreeing quite well with the slope of the phase sensitivity function (Fig. 1a). For a narrow phase distribution (γ ~ 0), the pulse had only a small influence. In contrast, for a wide phase distribution, the dark pulse provided a strong desynchronizing effect (Δ*R* ≈ −0.4 at γ ≈ 0.5 and Φ = 0.2 rad/2π) as well as a strong synchronizing effect (Δ*R* ≈ 0.3 at *γ* > 0.6 and Φ = 0.85 rad/2π). Sensitivity of the network dynamics to the dark pulse was, therefore, greater for the desynchronized state than for the synchronized state.

To destroy a strongly synchronized state, application of multiple pulses is quite efficient, as demonstrated in Fig. 1c. The synchronous activity was measured by the mean oscillation of all cells (
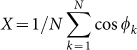
). After the application of three dark pulses around Φ = 0.2 rad/2π of the first three cycles, the synchronized state was weakened step by step. When the fourth dark pulse was applied at Φ = 0.2 rad/2π of the sixth cycle, the synchronized state was strongly suppressed, inducing the singularity behavior (upper panel of Fig. 1c). In contrast, when the fourth dark pulse was applied at Φ = 0.85 rad/2π of the sixth cycle, the synchrony was recovered (second panel of Fig. 1c). In the third and fourth panels of Fig. 1c, entrainments of the phase model to periodic applications of the dark pulse with a short cycle (23 h) and a long cycle (27 h) are shown. After some transient, the phase was locked to a stable fixed point, where synchronized dynamics of the cells was enhanced due to the entrainment and the mean oscillation *X* was amplified.

### Phase response curve for dark-pulse perturbation in *Arabidopsis thaliana*

**Figure 2:**
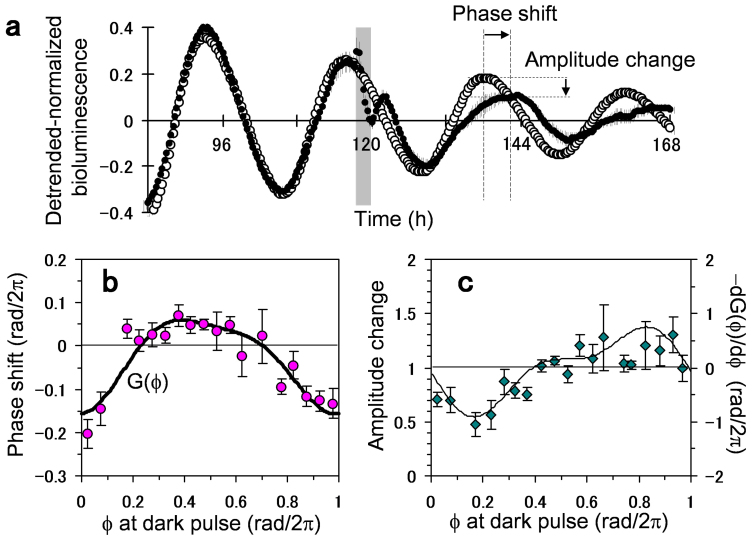
Circadian response of *Arabidopsis thaliana CCA1*::*LUC* to application of a 2-h dark pulse under constant light. (a) Bioluminescence signals were averaged over plants with (closed circle; n = 6) and without pulse perturbation (open circle; n = 40). The gray rectangle indicates the 2-h dark pulse. (b) Phase response curve for the dark pulse, indicating the phase shift of the plant circadian rhythm (n = 134) against phase Φ, at which the dark pulse was applied^24^. The solid line *G*(Φ) = −0.186 + 0.654 sin(Φ− 1.38) + 0.158 sin(2Φ − 1.669) shows a curve fitting using the first and second harmonics. (c) Amplitude response curve for the dark pulse, indicating the amplitude change of the plants (n = 169) against phase Φ of the pulse perturbation. The solid line represents −d*G*(Φ)/dΦ.

To apply our perturbation technique to a real plant system, we utilized a transgenic *Arabidopsis* strain *CCA1*::*LUC* carrying a firefly luciferase gene driven by a *CCA1* promoter, a pivotal circadian clock gene^22^. The bioluminescence signals were monitored by a Kondotron equipped with photomultiplier tubes^23^. As a pulse perturbation, 2-h dark pulses under constant light were applied. As shown in Fig. 2a, the dark pulses induced phase shifts and amplitude changes in the bioluminescence signal. In Fig. 2b, the PRC was drawn with respect to the circadian phase Φ, where Φ = 0 and π rad correspond to the subjective dawn and dusk, respectively. The PRC for the 2-h dark pulse was categorized as type-1 (characterized by a continuous transition from phase-delay to phase-advance without a breakpoint) and was fitted with a sinusoidal curve *G*(Φ)^24^. Normalized by the pulse duration of 2 h and shifted by its half duration of 1 h, the phase sensitivity function used in Eq. 1 is given by *Z*(Φ) = *G*(Φ − 2π/24)/2. Fig. 2c shows an amplitude response curve of the same plants. It can be seen that the dark pulse applied at Φ = 0.2 rad/2π (about the circadian time (CT) 7; noon) strongly suppressed the circadian oscillation, whereas it enhanced the circadian oscillation at Φ = 0.85 rad/2π (about CT 23; predawn). The effect of the pulse perturbation on the synchronized state depends upon the negative slope of the PRC^21^. If −d*G*(Φ)/dΦ < 0 or −d*G*(Φ)/dΦ > 2, the synchrony is weakened, whereas it is strengthened if 0 < −d*G*(Φ)/dΦ < 2. The negative slope of −d*G*(Φ)/dΦ was indeed consistent with the amplitude response curve (Fig. 2c).

### Singularity behavior and recovery of the circadian rhythm by dark-pulse perturbations

**Figure 3:**
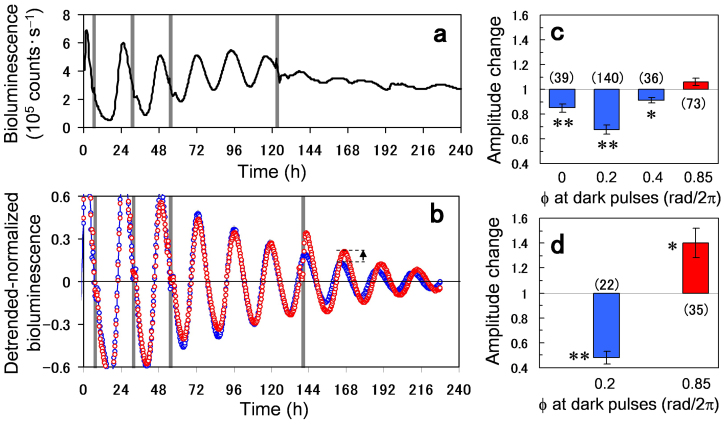
Amplitude modulations by multiple applications of 2-h dark pulse in *Arabidopsis thaliana CCA1*::*LUC*. (a) Four dark pulses marked by gray bars were applied to a plant at Φ = 0.2 rad/2π. Bioluminescence signal clearly indicates a singularity behavior. (b) Bioluminescence signal averaged over plants, to which first three pulses were applied at Φ = 0.2 rad/2π and fourth pulse was applied at Φ = 0.85 rad/2π (red circles; n = 35), and the one, to which first three pulses were applied at Φ = 0.2 rad/2π and no fourth pulse was applied (blue circles; n = 28). (c) Amplitude changes induced by the first three pulses applied at the phase of Φ = 0 or 0.2 or 0.4 or 0.85 rad/2π (n = 39, 140, 36, 73 for Φ = 0, 0.2, 0.4, 0.85; n = 37 for control without three pulses). (d) Amplitude changes induced by the fourth pulse applied at the phase of Φ = 0.2 or 0.85 rad/2π (n = 22, 35 for fourth pulse at Φ = 0.2, 0.85; n = 28 for control without fourth pulse). The significance of the pulse perturbation over the control condition was determined by Student's *t*-test (* P < 0.05, ** P < 0.01).

Next, we experimentally demonstrated the singularity behavior and recovery of the circadian rhythm as predicted by the phase oscillator model. Figure 3a shows the result of applying the 2-h dark pulse at Φ = 0.2 rad/2π for four times to a single intact transgenic seedling. The first three pulses gradually weakened the circadian oscillations and then the fourth pulse strongly suppressed the oscillation amplitude, clearly indicating the singularity behavior^25^. This singularity was observed in about 25% of the plant individuals used in our experiment (Fig. S1). Considering the delicate experimental procedure, which allows only a single trial of perturbation in each individual, this percentile is considered quite high. In contrast to the singularity experiment, when the first three pulses were applied at Φ = 0.2 and then the fourth pulse was applied at Φ = 0.85 rad/2π, the oscillation amplitude was recovered in Fig. 3b. Because of the damping effect, the amount of the recovered amplitude was less visible but it was significant. This implies that, depending upon the timing of the pulse injection, synchronous activities of the cellular oscillators can be either strengthened or weakened.

To clarify the effect of the first three pulses, we examined dependence of the perturbation on the phase, at which the three pulses were applied (Fig. 3c). After the three pulses were applied at either Φ = 0 or 0.2 or 0.4 or 0.85 rad/2π, the oscillation amplitude was measured at the fifth peak from the start of the measurement. The perturbed amplitude was then normalized by the one measured in a control experiment, in which no dark pulses were applied (n = 39, 140, 36, 73 for Φ = 0, 0.2, 0.4, 0.85; n = 37 for control). The amplitude change smaller (or larger) than 1 implies that the plant oscillation was weakened (or enhanced) by the dark pulses. Fig. 3c indicates that the three dark pulses decreased the circadian amplitude when they were applied at Φ = 0 or 0.2 or 0.4 rad/2π, while the amplitude was slightly increased when they were applied at Φ = 0.85 rad/2π. In Fig. 3d, we further examined the effect of the fourth pulse that followed the first three pulses. In this measurement, the first three pulses were applied at Φ = 0.2 rad/2π and then the fourth pulse was applied at either Φ = 0.2 or 0.85 rad/2π. The oscillation amplitude was measured at the first peak immediately after the fourth pulse (indicated by the arrow in Fig. 3b). The perturbed amplitude was then normalized by the one measured in a control experiment, in which the first three pulses were applied at Φ = 0.2 rad/2π and no fourth pulse was applied (n = 22, 35 for the fourth pulse at Φ = 0.2, 0.85; n = 28 for control without the fourth pulse). The fourth pulse applied at Φ = 0.2 rad/2π strongly weakened the oscillation amplitude, whereas the one applied at Φ = 0.85 rad/2π recovered the oscillation. The significance of the pulse perturbation over the control condition was determined by the Student's *t*-test (* P < 0.05, ** P < 0.01 in Fig. 3c and 3d).

### Spatiotemporal dynamics of cellular oscillators under dark-pulse perturbations

**Figure 4:**
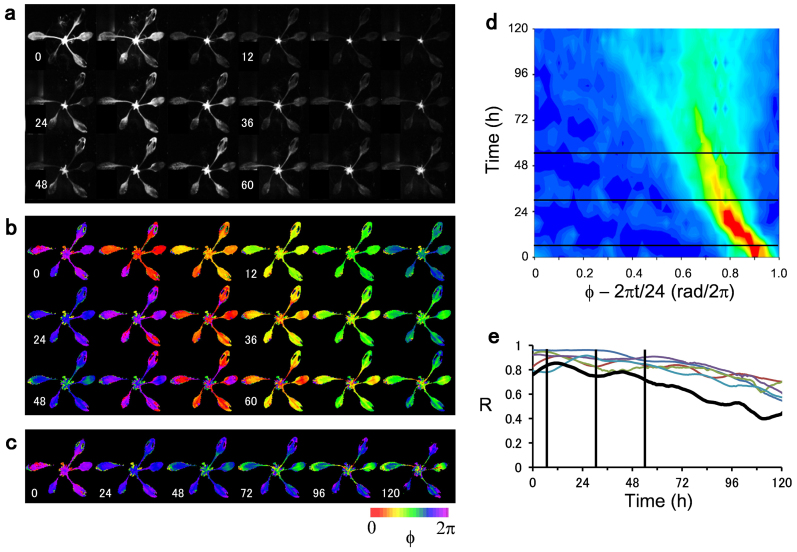
Spatiotemporal dynamics of cellular oscillators in a *CCA1*::*LUC* plant under continuous light. 2-h dark-pulses were applied at *t* = 6, 30, and 54 h. (a) Sequence of bioluminescence images of the plant shown with 4-h interval for 3 days duration. (b) Sequence of phase images corresponding to (a), where the phase Φ was computed from the bioluminescence signal. (c) Phase images with 24-h interval for 5 days duration. (d) Time evolution of the phase distribution of the whole plant shown in (b). Red and blue colors indicate high and low densities of the phase distribution, respectively. The black lines indicate the timings, at which the 2-h dark-pulses were applied. (e) Time evolution of the circular statistics *R*. The black line corresponds to *R* computed for the whole plant, whereas the non-black lines indicate *R* for individual leaves.

Now we visualize spatiotemporal dynamics of the cellular oscillators. For this recording, 2-h dark pulses were injected for three times (*t* = 6, 30, and 54 h) to a single intact transgenic seedling. Bioluminescence images of the plant composed of five leaves are sequentially displayed with 4-h interval in Fig. 4a. The corresponding phase images of the bioluminescence signal indicate clear desynchronized activities of the plant circadian oscillators in both cellular level and leaf level (Fig. 4b, c). Within each leaf, phase wave propagations as well as phase delays caused by the vein network are discernible as reported in ref. 6. The desynchronized activities are even more pronounced among the leaves. Namely, initially synchronized activities between the leaves were eventually separated and a clear anti-phase relationship appeared after *t* = 72 h (Fig. 4c). The phase-time plot of Fig. 4d indicates that the phase distribution was rapidly broaden by the dark-pulses. A population of strongly coherent cell activities colored in red and yellow was diffused and disappeared after *t* = 24 h, while the range of phase distribution was expanded from 0.2 rad/2π (*t* = 6 h) to 0.6 rad/2π (*t* = 120 h). To quantify the level of synchrony, the circular statistics, *R*, were computed in Fig. 4e for the whole plant (black line) and for the individual leaves (non-black colors). Although the synchronous cellular activities were maintained to some extent within a leaf level, the whole plant showed a lowered level of synchrony. This implies that the circadian cells interact mainly with their nearby cells and that the effect of interaction becomes weak between the distant leaves. The first two dark pulses applied at *t* = 6, 30 h had a relatively weak effect on the cellular rhythms compared to the third pulse applied at *t* = 54 h, which strongly destroyed the coherent cellular activities and accelerated their transition to desynchrony. This is in good agreement with our modeling study, which indicates that the pulse had a stronger influence on loosely synchronized cells rather than on tightly synchronized cells (Fig. 1b). In fact, main features of the phase-time plot of Fig. 4d can be well reproduced by our model (Fig. S5).

### Entrainments to periodically applied dark-pulses

**Figure 5:**
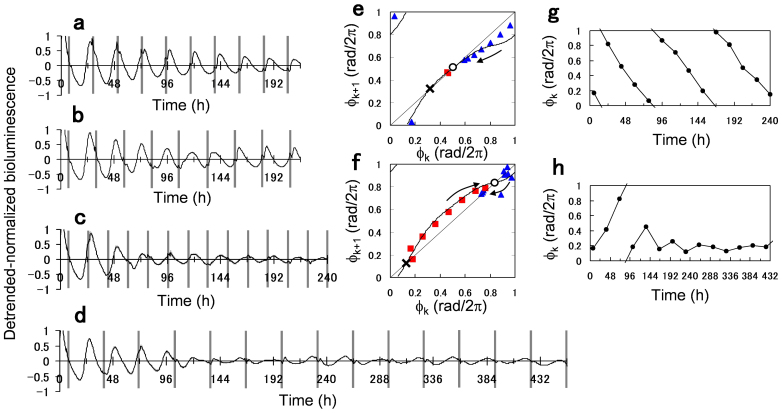
Entrainment to periodically applied 2-h dark pulses. (a–d) Bioluminescence signals of the *CCA1*::*LUC* plant perturbed by 2-h dark pulses with a period of (a) *T* = 22 h, (b) *T* = 25 h, (c) *T* = 18 h, and (d) *T* = 32 h. Each panel represents the averaged signal over *n* = 30, 22, 20 and 9 plants, respectively. Gray bars indicate timing of 2-h dark pulses. (e, f) Return maps of phase Φ_k_ observed at the *k*th dark pulse under *T* = 22 h (e) and 25 h (f). The points were obtained from (a) and (b). The red squares and blue triangles indicate transient processes to approach the stable fixed point with increasing and decreasing direction of Φ_k_, respectively. The solid lines indicate Φ_k+1_ = Φ_k_ + G(Φ_k_) + 2π(T − τ)/τ mod 2π with a natural period of τ = 23 h. The open circle and cross represent stable and unstable fixed points, respectively, obtained as solutions of G(Φ) + 2π(T − τ)/τ = 0. (g, h) Time series of the phase Φ_k_ under (g) *T* = 18 h and (h) *T* = 32. The points were obtained from (c) and (d).

As a final experiment, we demonstrated the entrainment of the plant circadian rhythm to periodic application of dark pulses with various periods *T*. First, the plant was entrained to periodic dark pulses with *T* = 22 and 25 h (Figs. 5a and 5b). To analyze the phase locking process, Figs. 5e and 5f show the return maps between consecutive phases Φ_k_ and Φ_k+1_, where Φ_k_ represents the circadian phase when the *k*th dark pulse was applied. After some transient, the phase was locked to a stable fixed point of about Φ = 0.5 and 0.75 rad/2π for *T* = 22 and 25 h, respectively. Assuming synchronized activities of the cellular rhythms, the phase dynamics of the plant is reduced to a single oscillator model of Φ_k+1_ = Φ_k_ + G(Φ_k_) + 2π(T − τ)/τ, where τ represents a natural period of the circadian rhythm (τ = 23 h in the present experiment). Stable and unstable fixed points can be estimated as solutions of G(Φ) + 2π(*T* − τ)/τ = 0, where the stable fixed point is located in a negative region of d*G*(Φ)/dΦ. The location of the stable fixed point obtained by the phase model agrees quite well with the one observed experimentally (open circle). Moreover, the transient process of decreasing or increasing Φ_k _can be well traced along the return map (solid line of Figs. 5e and 5f).

We then applied the periodic dark pulses with period *T*, which is significantly different from the natural period τ. For the very short period (*T* = 18 h), the phase Φ_k_ decreased linearly and the entrainment did not occur (Figs. 5c and 5g). On the other hand, for the very long period (*T* = 32 h), the phase Φ_k_ increased linearly at the beginning, but later locked to a constant value (Figs. 5d and 5h). We note that this adaptation phenomenon is slightly different from the usual entrainment in the sense that the circadian amplitude does not grow much compared to that of the usual entrainment, which would amplify the rhythm much more strongly. Importantly, the present results including the adaptation phenomenon can be reproduced quite well by the phase-oscillator model of Eq. 1 (shown in Figs. 1c and S4).

## Discussions

We presented a method for controlling plant circadian rhythms using dark pulses under constant light. The methodology was based upon the phase modeling of weakly coupled circadian oscillators, which was proven precise enough to engineer delicate synchronization features of the plant system. Our method does not require detailed physical, chemical, or biological knowledge of the system but utilizes only a measurement of PRC and a small amount of pulse perturbations, which do not destroy fundamental properties of the plant. It should find many applications in complex engineering problems such as the design of pacemakers as well as antipacemakers for biological systems for which detailed models are difficult to determine^26^.

The significance of our method was demonstrated by the singularity behavior induced in the plant circadian rhythm. The circadian singularity was first reported by Winfree (1970)^27^ who analyzed phase resetting data of *Drosophila pseudoobscura* in response to a single discrete perturbation and found that a unique stimulus time and duration strongly altered the phase resetting. The singularity or circadian arrhythmia was then observed in many other organisms including *Kalanchoë* flowers^28^, humans^29^, *Chlamydomonas*^30^, and Djungarian hamsters^31^. There have been mainly two theoretical models to elucidate the singularity behavior. The first one is based upon circadian desynchronization, where the singularity is attributed to diversification of phases within the population of circadian oscillators^16^. The second one is based upon a limit cycle model, where a strong stimulus suppresses the amplitude of individual circadian oscillators and leads them to a phaseless state^15^,^16^,^32^,^33^,^34^. The desynchronization model was validated by a synthetic system for mammalian cellular clocks^35^, whereas the effect of amplitude suppression was confirmed in *Neurospora*^36^ as well as in SCN tissue data of Siberian hamsters^37^. Our study is along the line of the desynchronization model in the sense that the plant system was controlled in such a way to destroy the synchronized cellular activities. Our results however do not exclude the model of the amplitude suppression, because the damping of the oscillation amplitude, as seen in the slowly weakened rhythms of Figs. 2a, 3a, and 3b, is an inherent property of the plant system. The two models may contribute to the singularity behavior of the plant in a complementary manner.

Although the phase oscillator model was good enough to predict the key features of controlling the plant circadian system, we should note its limitations. As discussed above, the plant system produces weakly damped oscillations under constant light. The dark pulse affects not only phase but also, to some extent, amplitude of the plant rhythm. A refined model, which takes into account both phase and amplitude of damped oscillators, *e.g.*, coupled Stuart-Landau equations^6^, may further improve the design accuracy of our control technique.

In plant, cell-to-cell communications are established through two major connections: plasmodesmata and vascular bundles. Plasmodesmata directly connect nearest-neighbor cells, whereas vascular bundles spread over the whole plant body to rapidly transport materials among all tissues. In this sense, the plant circadian system has properties of both local and global connections. Moreover, due to inactive cells in the vein region, the network of circadian cells in leaves shows various patterns of phase dynamics as well as phase delays^6^,^8^,^9^. Taking into account such inactive regions with more detailed network topology might further improve our model.

## Methods

### Plant growth and bioluminescence monitoring conditions

*CCA1::LUC* plants were grown on 4 ml of an gellan gum-solidified Murashige and Skoog plant salt mixture with 2% (w/v) sucrose in a 40-mm dish under 12 h light (100 μmol m^−2^ s^−1^ of fluorescent white light): 12-h dark cycles at 22 ± 0.5°C for 14 days. About 24 h before the start of the bioluminescence monitoring, the plants were treated with 500 μl of luciferin (1 mM) dissolved in water. The bioluminescence assays were carried out using a monitoring system, known as *Kondotron*, developed by Kondo *et al.* (1993)^23^,^38^. Using this system, the bioluminescence was detected for each plate by a photomultiplier tube (PMT) (Hamamatsu H7360-01MOD; Hamamatsu Photonics KK, Japan) enclosed in a light-tight box. Each plate was on a turntable, which was located under the PMT and rotated sequentially every 20 min under the control of a computer. On each plate, the plant was exposed to darkness for 4 min every 20 min (at least 1.5 min of darkness is needed to allow chlorophyll fluorescence decay). Such a brief exposure to darkness does not have a major effect on the circadian rhythms in plants^4^ or in cultured cells^22^. The bioluminescence monitoring system was placed in a temperature-controlled chamber (MIR-553, SANYO Electric Co. Ltd.) at 22 ± 0.5°C. Light-dark conditions were generated using red LEDs (λ_p_ = 660 nm, 100 μmol m^−2^ s^−1^) controlled by a computer. The recorded bioluminescence data were detrended by applying the moving average filter (24-h window size) twice and were then normalized.

### Numerical simulations

In the phase oscillator model of Eq. 1, the phase sensitivity function *Z*(Φ) for the dark pulse is given by *Z*(Φ) = *G*(Φ − 2π/24)/2, where *G*(Φ) represents PRC measured in Fig. 2b. By the least squares method, the PRC was approximated as *G*(Φ) = −0.186 + 0.654 sin(Φ− 1.38) + 0.158 sin(2Φ − 1.669) using the first and second harmonics as (24) and then used for the model simulations. Natural frequencies {ω_k_} of the model were considered to be normally distributed, *i.e.*, ω_k_∈*N*(<ω>,σ_ω_). In the numerical studies of Fig. 1c, the parameters were set to be *K* = 0.01, <ω> = 2π/24 h^−1^, and σ_ω_/<ω> = 0.05. The coupling strength was determined to fit to the experimental data (Fig. S2). For simplicity, no coupling (*K* = 0) and identical oscillators (ω_k_ = 2π/24 h^−1^) were assumed in Fig. 1b.

### Data processing

#### Phase definition

To extract phase information from the bioluminescence signals, the phase was computed as follows. To draw the PRC of Fig. 2b and the phase image in Fig. 4, the phase was defined as Φ(t) = 2π(t − τ_k_)/(τ_k+1_ − τ_k_), where τ_k_ represents the time at which the signal took its *k*th peak and τ_k_ < = *t* < τ_k+1_^6^. This approach provides a reliable phase estimate if the bioluminescence signal is not noisy and the peak picking works efficiently. For periodically modulated signals of Fig. 5, which sometimes shows unclear structure of the peaks, the phase was extracted from the detrended and normalized bioluminescence signal through the Hilbert transform.

## Author Contributions

H.F. designed the project; H.F. and H.M. carried out the experiments; H.F. and I.T.T. simulated the model and analyzed the data; and H.F. and I.T.T. wrote the manuscript. All authors discussed the results and commented on the manuscript.

## Supplementary Material

Supplementary Information Supplementary Information
