# Supplementary material for: Controlling Circadian Rhythms by Dark-Pulse Perturbations in Arabidopsis thaliana
Source: Sci Rep. 2013 Mar 25;3:1533. doi: 10.1038/srep01533 (PMC3607175; doi:10.1038/srep01533)
Supplement: Supplementary Information [file srep01533-s1.pdf]

Supplementary Information for

Controlling Circadian Rhythms by Dark-Pulse Perturbations in *Arabidopsis thaliana*

Hirokazu Fukuda[1,2]*, Haruhiko Murase[1] & Isao T. Tokuda[3]

[1] Department of Mechanical Engineering, Graduate School of Engineering,
Osaka Prefecture University, Sakai 599-8531, Japan
[2] PRESTO, Japan Science and Technology Agency (JST),
4-1-8 Honcho Kawaguchi, Saitama 332-0012, Japan
[3] Department of Mechanical Engineering, Ritsumeikan University,
1-1-1 Nojihigashi, Kusatsu, Shiga 525-8577, Japan

* Correspondence to: fukuda@me.osakafu-u.ac.jp

S1. Singularity behaviors induced by 2-h dark pulses in *Arabidopsis thaliana CCA1::LUC*.
S2. Determination of the coupling strength *K* in Eq. 1 based on computer simulations.
S3. Numerical simulation of phase distributions before and after pulse perturbation.
S4. Numerical simulation of phase oscillators entrained to periodic dark pulses with a very long cycle.
S5. Time evolution of the phase distribution in globally coupled phase oscillators (Eq. 1).

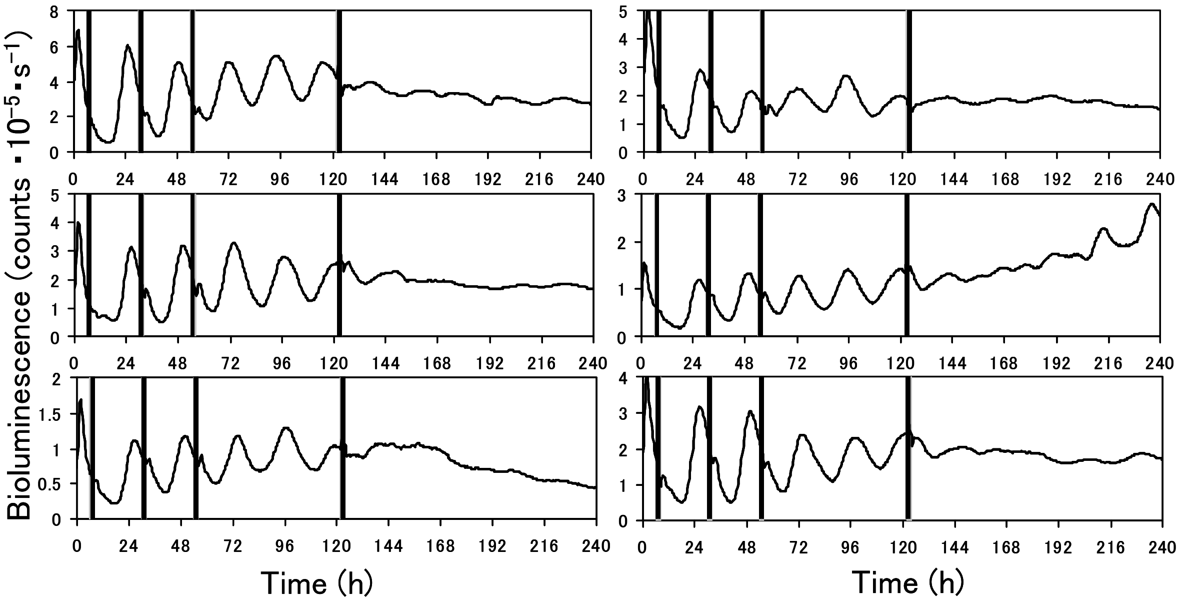

Figure S1. Singularity behaviors induced by 2-h dark pulses in *Arabidopsis thaliana CCA1::LUC*. In the same procedure as in Fig. 3a, four pulses were applied to 6 different plants to weaken the circadian rhythm. After the first three pulses, the fourth pulse strongly suppressed the rhythm and induced the singularity behavior. Among 23 plants, this type of singularity was clearly observed in the present 6 plants.

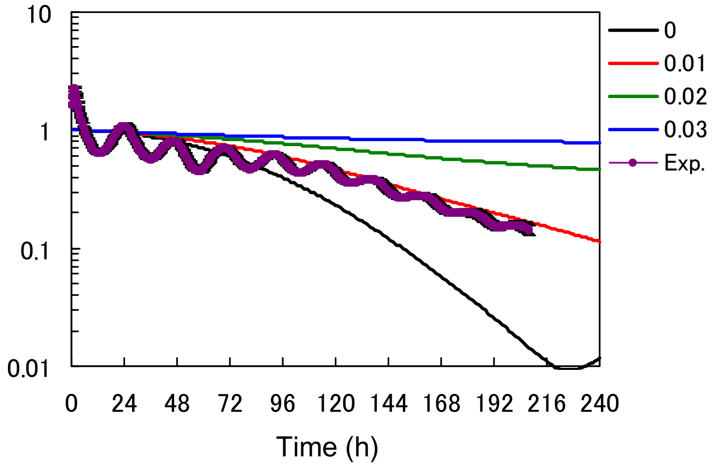

Figure S2. Determination of the coupling strength *K* in Eq. 1 based on computer simulations. Time-dependent profile of the circular statistic, *i.e.*, synchronization index *R*, was drawn for *K* = 0, 0.01, 0.02 and 0.03. The profile was also drawn for the experimental data of the amplitude change, in which the free-running plant rhythm was recorded without dark-pulse perturbation. The amplitude data were extracted from the bioluminescence signals through the Hilbert transform. Since the coupling strength of *K* = 0.01 was found to show the profile most similar to the experimental one, *K* = 0.01 was used in our simulation study.

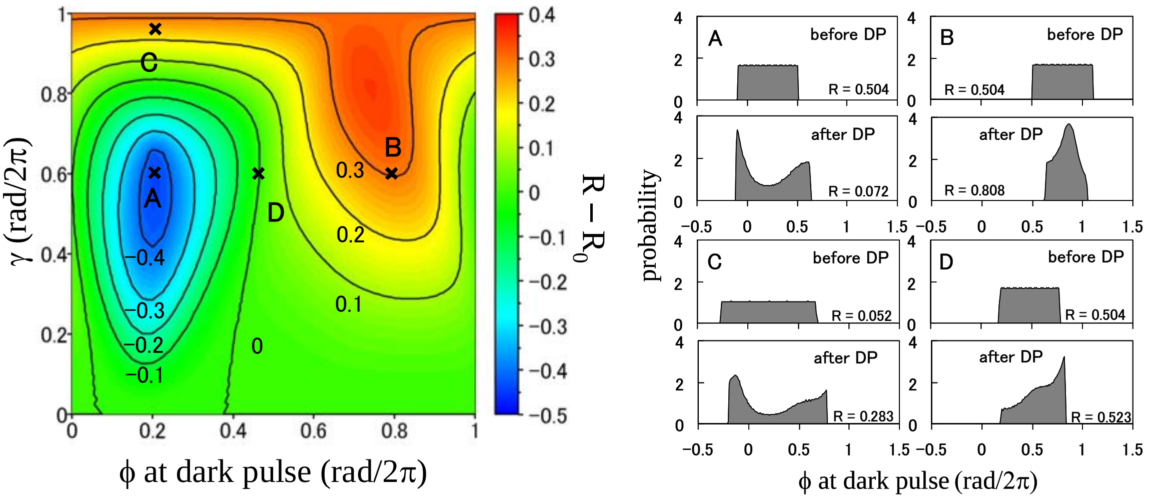

Figure S3. Numerical simulation of phase distributions before and after pulse perturbation.

Left panel corresponds to enlargement of Fig. 1b, which shows the dependence of the effect of pulse perturbation on the mean ϕ and range γ of the uniformly distributed phase of the oscillators $[\phi - \gamma, \phi + \gamma]$. At the four points (A, B, C, D) indicated by crosses on the left panel, phase distributions before and after application of 2-h dark pulse are compared on the right panel.

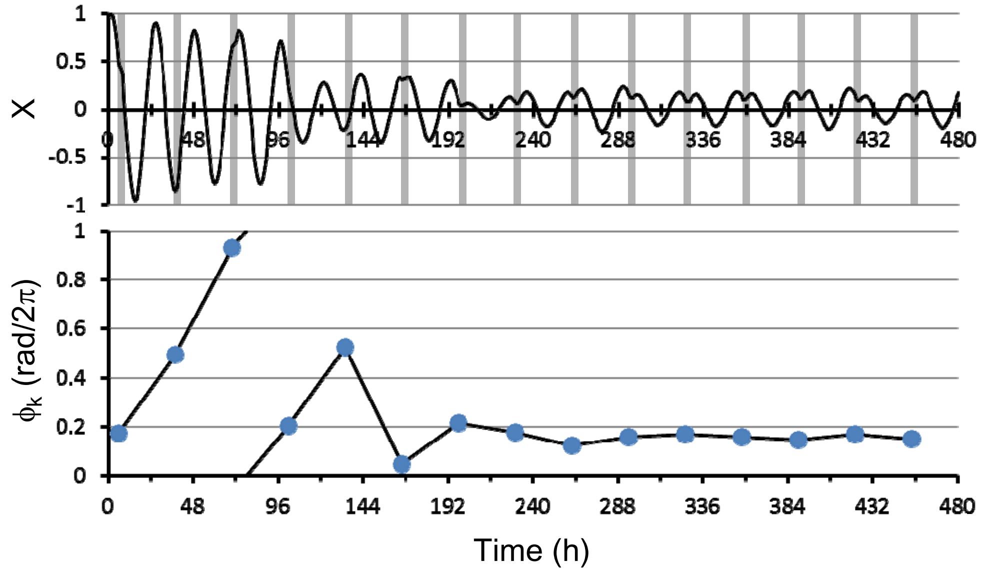

Figure S4. Numerical simulation of phase oscillators entrained to periodic dark pulses with a very long cycle.

(upper panel) Mean oscillation amplitude X in globally coupled phase oscillators (Eq. 1) under periodic dark pulses with T = 32 h. Gray bars indicate timing of 2-h dark pulses. (lower panel) Time series of the phase ϕ_k of X observed at the kth dark pulse on the upper panel. The parameters were set to be $K = 0.01$, $\langle\omega\rangle = 2\pi/23\ h^{-1}$, and $\sigma_\omega/\langle\omega\rangle = 0.05$.

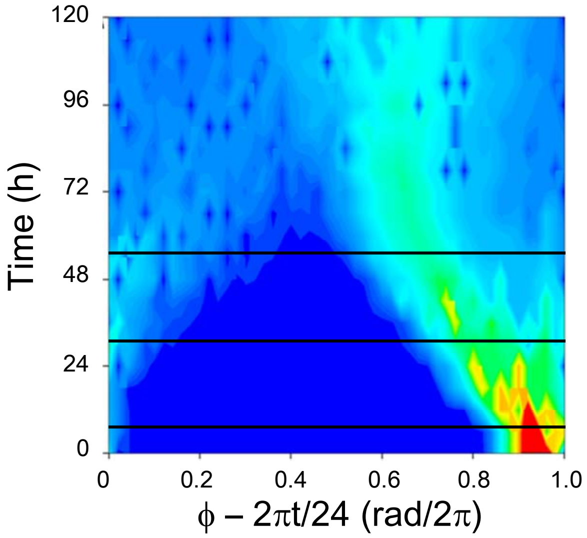

Figure S5. Time evolution of the phase distribution in globally coupled phase oscillators (Eq. 1). Red and blue colors indicate high and low densities of the phase distribution, respectively. The black lines indicate the timings, at which the 2-h dark-pulses were applied. In this figure, the phase was redefined as $\phi(t) = 2\pi(t - \tau_k)/(\tau_{k+1} - \tau_k)$ from the peak times τ_k of the signal $x_i = \cos(\phi_i)$ to compare to experimental data (Fig. 4d). The parameters were set to be $N = 20000$, $K = 0.01$, $\langle\omega\rangle = 2\pi/23$ h^{-1}, and $\sigma_\omega/\langle\omega\rangle = 0.05$.
